# Risk of pulmonary embolism and deep vein thrombosis in systemic lupus erythematosus: a population-based cohort study

**DOI:** 10.1186/ar3987

**Published:** 2012-09-27

**Authors:** JA Aviña-Zubieta, D Lacaille, EC Sayre, J Kopec, HK Choi, JM Esdaile

**Affiliations:** 1 Arthritis Research Centre of Canada, University of British Columbia, Vancouver, BC, Canada; 2 University of British Columbia, Vancouver, BC, Canada; 3 Boston University School of Medicine, Boston, MA, USA; 4 Harvard School of Public Health, Boston, MA, USA

## Background

A recent hospital-based study suggested a 10-fold increased risk of pulmonary embolism among individuals with systemic lupus erythematosus (SLE) in the year following hospital admission. It is unknown whether the risk is similar among the nonhospitalized SLE population. We estimated the risk of incident pulmonary embolism (PE) and deep venous thrombosis (DVT) events, as well as the associated time trend, among incident cases of SLE compared with general population controls using physician billing and hospitalization data for the entire province of British Columbia, Canada (~5 million).

## Methods

Our data included all visits to health professionals and hospital admissions covered under the province's universal healthcare plan from 1 January 1990 until 31 December 2007 for all individuals ≥18 years of age. We conducted a matched cohort study among patients meeting the following criteria: ≥18 years of age, and new diagnosis of SLE based on the following algorithm: one ICD code for SLE on rheumatologist visit billing data or on hospitalization data, or two ICD codes for SLE at least 2 months and no more than 2 years apart on a physician visit by a nonrheumatologist. Controls were selected from the general population, on a 10:1 ratio for each case, matched by birth year, sex and calendar year of exposure. The outcomes, PE and DVT, we identified based on one ICD code for PE in hospitalization data; and one ICD code for DVT in either outpatient or hospitalization data. We estimated relative risks (RRs) of PE and DVT in SLE cases compared with matched general population controls, after adjusting for age, sex, comorbidities, trauma, fracture, surgery, and hospitalizations.

## Results

Among 5,156 individuals with SLE, 54 developed PE and 92 developed DVT. Compared with age-matched, sex-matched, and entry-time-matched controls (*n *= 51,560), the RRs were 4.9 (95% CI = 3.4 to 6.8) for PE and 4.5 (95% CI = 3.5 to 5.7) for DVT. These RRs attenuated slightly after adjusting for covariates, but remained significant (Table 1). When we evaluated the impact of follow-up time, the RRs for PE, DVT and PE or DVT in SLE patients as compared with non-SLE cases were the largest in the first year (Table 2). The estimates decreased over time and were not significant after 5 years of follow-up with the exception of DVT.

**Table 1:** 

	SLE	Non-SLE
PE		
Cases (*n*)	54	114
Incidence rate/1,000 person-years	2.5	0.5
Age-, sex-, entry-time-matched RR (95% CI)	4.9 (3.4 to 6.8)	1.0
Multivariable RR (95% CI)	4.6 (3.3 to 6.4)	1.0
DVT		
Cases (*n*)	92	214
Incidence rate/1,000 person-years	4.3	1.0
Age-, sex-, entry-time-matched RR (95% CI)	4.5 (3.to 5.7)	1.0
Multivariable RR (95% CI)	4.1 (3.2 to 5.2)	1.0
PE or DVT		
Cases (*n*)	131	295
Incidence rate/1,000 person-years	6.2	1.3
Age-, sex-, entry-time-matched RR (95% CI)	4.7 (3.8 to 5.7)	1.0
Multivariable RR (95% CI)	4.3 (3.5 to 5.3)	1.0

Relative risk of incident PE and DVT according to SLE status

**Table 2:** Incidence rates and relative risks for PE, DVT and PE or DVT in patients with SLE during follow-up

	<1 year	1 to 5 years	>5 years of follow-up
PE events	31	20	3
DVT events	45	36	11
PE or DVT events	70	49	12
Incidence rate of PE/1,000 person-years	6.7	1.6	0.6
Incidence rate of DVT/1,000 person-years	9.8	3.0	2.3
Incidence rate of PE or DVT/1,000 person-years	15.5	4.1	2.6
Age-, sex-, entry-time-matched RR of PE (95% CI)	18.4 (9.9 to 35.5)	3.2 (1.8 to 5.3)	1.0 (0.2 to 3.1)
Age-, sex-, entry-time-matched RR of DVT (95% CI)	10.2 (6.6 to 15.8)	3.0 (2.0 to 4.4)	2.5 (1.2 to 5.0)
Age-, sex-, entry-time-matched RR of PE or DVT (95% CI)	13.6 (9.4 to 19.8)	3.0 (2.2 to 4.2)	1.7 (0.9 to 3.2)

## Conclusion

This is the first population-based study assessing the risk of PE and DVT in patients with SLE. These findings support increased monitoring of venous thromboembolic complications and risk factors in SLE patients, especially during the first year after disease onset.

